# Supplementary material for: Long Non-Coding RNAs Associated with Mitogen-Activated Protein Kinase in Human Pancreatic Cancer
Source: Cancers (Basel). 2023 Jan 2;15(1):303. doi: 10.3390/cancers15010303 (PMC9818929; doi:10.3390/cancers15010303)
Supplement: Supplementary file 1 [file cancers-15-00303-s001.zip › Supplementary Figures.pdf]

Methodological flowchart

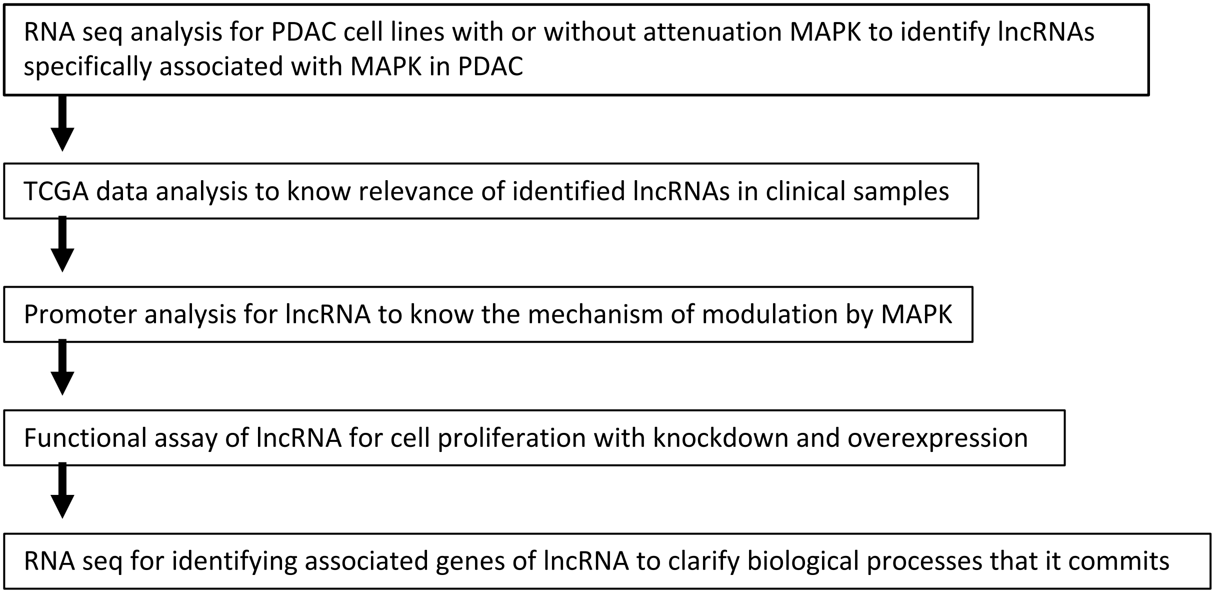

Supplementary Figure S1. Methodological flowchart of this study.

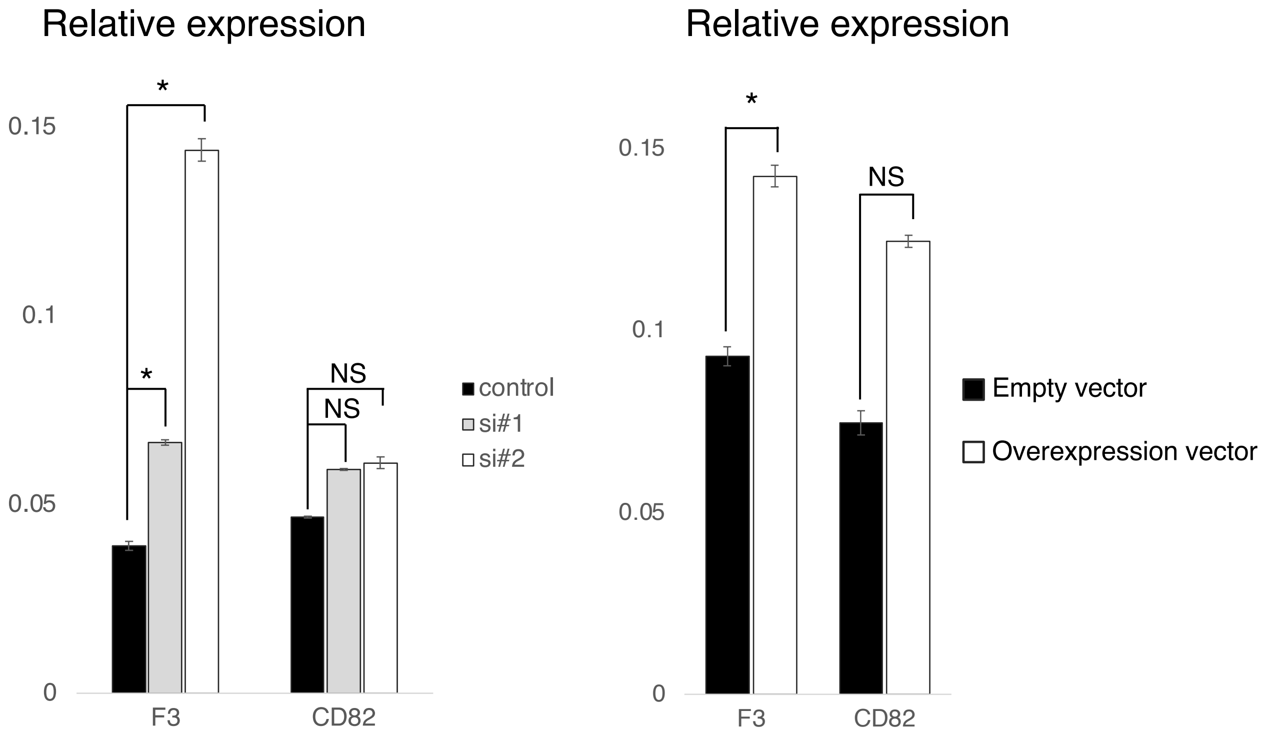

Supplementary Figure S2. qRT-PCR of *LINC00941-associated* genes in AsPC-1.

Both the downregulation and upregulation of *LINC00941* resulted in the upregulation of *F3* and *CD82*, which suggested *LINC00941* does not directly regulate them.

Abbreviation; NS, no significance. **p<0.05*

(A)

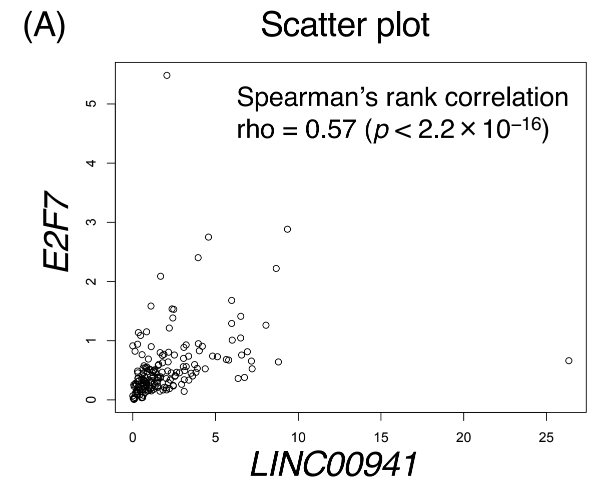

(B)

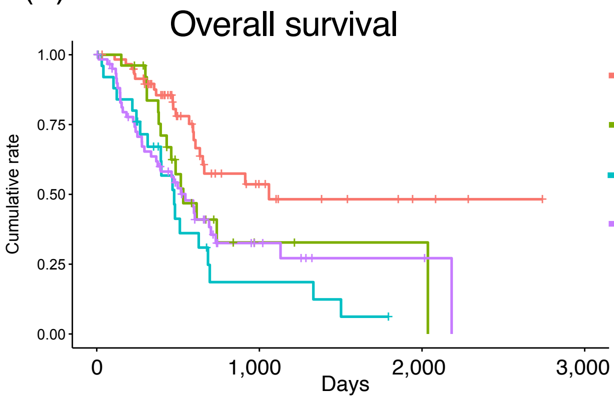

LINC00941	*E2F7*	Cases
low	low	62
low	high	26
high	low	26
high	high	61
	Total:	175

Log-rank test

	low/high	high/low	high/high
low/low	$p = 0.04$	$p = 3 \times 10^{-4}$	$p = 0.002$
low/high	-	$p = 0.1$	$p = 0.5$
high/low	-	-	$p = 0.4$

Supplementary Figure S3. Correlation between LINC00941 and E2F7 based on TCGA database analysis. (A) A positive correlation was observed between gene expression levels of *LINC00941* and *E2F7*. (B) Kaplan-Meier plot comparing the overall survival of the groups classified based on the gene expression level of *LINC00941* and *E2F7*. The low/low group showed a better prognosis than others.
